# Molecular biomarkers in metastatic clear cell renal cell carcinoma treated with first-line immune combinations: a systematic review of phase III randomized clinical trials by Meet-URO group

**DOI:** 10.1007/s10555-026-10371-w

**Published:** 2026-08-26

**Authors:** Anna Amela Valsecchi, Michele Maffezzoli, Maria Concetta Cursano, Fabrizio Di Costanzo, Andrea Malgeri, Mattia Alberto Di Civita, Brigida Maiorano, Carlo Messina, Giuseppe Procopio, Davide Bimbatti, Sebastiano Buti, Sara Elena Rebuzzi, Massimo Di Maio

**Affiliations:** 1 Department of Oncology, University of Turin, A.O.U. Città Della Salute E Della Scienza Di Torino, Ospedale Molinette, Turin, Italy; 2 Medical Oncology Unit 2, Ospedale Molinette, A.O.U. Città Della Salute E Della Scienza Di Torino, Ospedale Molinette, Turin, Italy; 3 Oncology Unit, University Hospital of Parma, Parma, Italy; 4 Department of Oncology, IRCCS Istituto Romagnolo Per Lo Studio Dei Tumori (IRST) Dino Amadori, Meldola, Italy; 5 Translational and Clinical Research Institute, Newcastle University Centre for Cancer, Newcastle Upon Tyne, UK; 6 Department of Medical Oncology, Fondazione Policlinico Universitario Campus Bio-Medico, Rome, Italy; 7 Department of Experimental Medicine, Sapienza University of Rome, Rome, 00161 Italy; 8 Department of Medical Oncology, IRCCS San Raffaele Hospital, Milan, Italy; 9 Oncologu Unit, ARNAS Civico Benfratelli Di Cristina, Palermo, Italy; 10 Department of Oncology, Istituto Nazionale Tumori Milano, Milan, Italy; 11 Oncology 1 Unit, Istituto Oncologico Veneto, IOV - IRCCS, Padua, Italy; 12 Medicine and Surgery Department, University of Parma, Viale Antonio Gramsci, 14, 43126 Parma, Italy

**Keywords:** Clear cell renal cell carcinoma, Biomarkers, Molecular, Immunotherapy, First line

## Abstract

Immune checkpoint inhibitor (ICI)–based combinations represent the standard first-line treatment for metastatic clear cell renal cell carcinoma (mRCC), although robust biomarkers for treatment selection remain undefined. We conducted a systematic review to identify biomarkers assessed in randomized clinical trials (RCTs) on first-line ICI-based regimens. Following PRISMA guidelines, we searched PubMed, Web of Science and Scopus (January 2018–October 2025) for phase III RCTs investigating first-line ICI-based therapies in mRCC with molecular or circulating biomarker analyses. Given heterogeneity across studies, a qualitative synthesis was performed. Sixteen reports were included. Biomarkers were assessed using immunohistochemistry, transcriptomics, genomic sequencing and blood analyses. PD-L1 expression did not reliably discriminate benefit across ICI-based combinations, although it revealed a negative prognostic influence with sunitinib and a potential predictive role for nivolumab-ipilimumab. Tumours with angiogenic signatures were consistently associated with improved outcomes, suggesting prognostic relevance, while derived limited additional benefit from ICIs. Immune-related signatures were associated with ICI response, whereas proliferation and *MYC*-related signatures identified disease with poor prognosis across treatments. Individual mutations (e.g. *PBRM1*, *VHL*, *BAP1*, *PTEN*) showed heterogeneous and mainly prognostic associations, whereas composite gene panels (e.g. rDM) may offer better predictive value. Circulating biomarkers, including inflammatory cytokines and KIM-1 dynamics, demonstrated promising prognostic and early predictive signals. No validated biomarker currently supports treatment selection among first-line ICI-based combinations in mRCC. Future research should prioritize adaptive, biomarker-guided trial designs integrating longitudinal multi-omic profiling and circulating biomarkers to generate clinical evidence and support personalized treatments.

**Supplementary Information:**

The online version contains supplementary material available at 10.1007/s10555-026-10371-w.

## Introduction

Over the past decade, the therapeutic landscape of first-line metastatic renal cell carcinoma (mRCC) has shifted from single-agent tyrosine kinase inhibitors (TKIs) targeting the vascular endothelial growth factor (VEGF) pathway toward immune checkpoint inhibitor (ICI)–based combinations, including dual immunotherapy or ICI plus TKI regimens [1]. Despite these advances, substantial interpatient variability in treatment outcomes persists, highlighting both the unmet need for predictive and prognostic biomarkers capable of guiding personalized therapeutic strategies and the requirement to elucidate mechanisms of resistance. In this context, mRCC represents a biologically heterogeneous disease with variable clinical behaviour and response to systemic therapies.

Clear cell renal cell carcinoma (ccRCC), the most common histological subtype of mRCC, is defined by recurrent genomic alterations that affect chromatin remodelling, hypoxia signalling, and immune regulation. Loss of function of the von Hippel–Lindau (*VHL*) tumour suppressor gene is an early and nearly universal event in ccRCC tumorigenesis, resulting in constitutive stabilization of hypoxia-inducible factors (HIFs) and upregulation of angiogenic pathways, including VEGF, PDGF, and TGF-α, which drive tumour vascularization and progression [2, 3]. Beyond *VHL*, frequent mutations in chromatin-modifying genes such as *PBRM1*, *BAP1*, *SETD2* and *KDM5C* define distinct molecular subgroups associated with differential tumour aggressiveness, metastatic potential and clinical outcomes [4, 5]. For instance, *PBRM1* mutations are generally linked to a less aggressive phenotype, whereas *BAP1* alterations correlate with high-grade tumours and poor prognosis [6]. Early biomarker studies investigating programmed death ligand 1 (PD-L1) expression and tumour mutational burden (TMB) demonstrated inconsistent predictive value for immunotherapy response, highlighting the limitations of single-parameter biomarkers [7]. Similarly, the International mRCC Database Consortium (IMDC) risk classification, while initially developed to guide VEGF-targeted therapy, has shown limited reliability in predicting response to ICIs [8, 9]. These findings underscore the pressing need to identify prognostic and predictive biomarkers which could enable personalized therapeutic strategies in mRCC.

Many biomarker analyses have been conducted, but with heterogeneous results, so that no molecular biomarker has yet achieved sufficient clinical validity to be implemented routinely in treatment decision-making. The aim of the present systematic review is to comprehensively evaluate the existing literature on molecular and blood biomarkers assessed in mRCC, with an update focus on their predictive and prognostic value in first-line combinations from randomized trials. This review seeks to clarify the current role of molecular profiling in guiding first-line treatment selection and to identify knowledge gaps for future research.

## Materials and methods

**Fig. 1:**
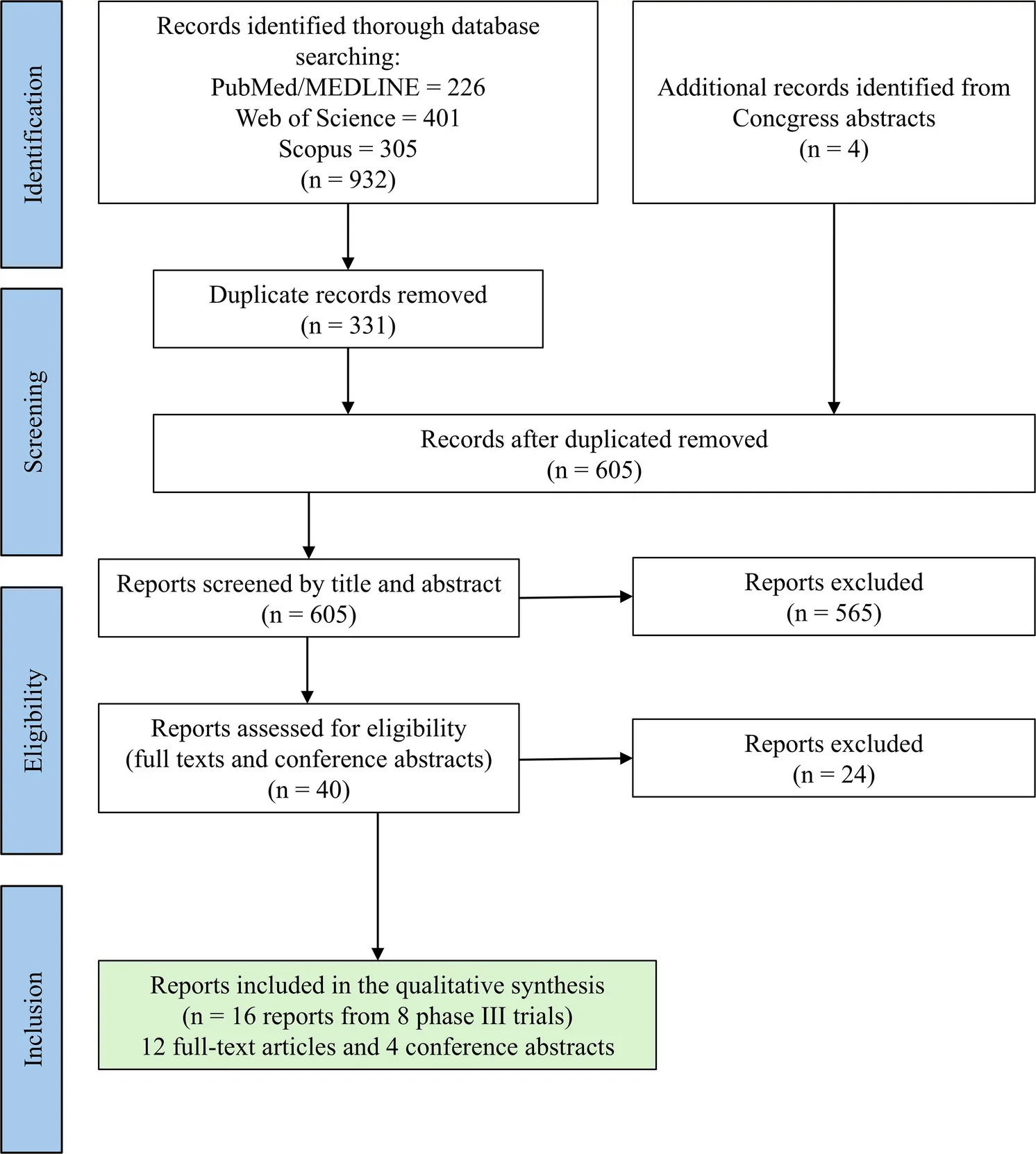
Preferred Reporting Items for Systematic Reviews and Meta-Analyses (PRISMA) diagram

This systematic review was registered in the International Prospective Register of Systematic Reviews (PROSPERO; registration number CRD420261451337) and conducted according to the Preferred Reporting Items for Systematic reviews and Meta-Analyses (PRISMA) guidelines, as reported in Fig. 1. Full information on search details, study-selection counts and reasons for reports exclusion are reported in Supplementary Table 1.

### Eligibility criteria

Eligibility was assessed at the report level for each individual publication or conference abstract, while the underlying study was required to be a phase III randomized trial.

Reports were eligible if they met all of the following criteria: (1) they presented data from a phase III randomized trial evaluating first-line ICI-based treatments for mRCC; (2) the study population included adult patients with metastatic ccRCC receiving first-line systemic treatment; (3) at least one randomized treatment arm evaluated an ICI-based combination, including dual immune checkpoint inhibition, an ICI plus TKI or an anti-angiogenic agent; (4) they reported an original biomarker analysis, either pre-specified or exploratory, performed on tumour tissue or peripheral blood samples; and (5) they evaluated the association between the biomarker and clinical outcomes and/or treatment effect.

Eligible reports included primary trial publications, secondary or post hoc biomarker analyses and conference abstracts providing biomarker data (sufficient to be extracted) and published between 1 January 2018 and 18 October 2025. Multiple reports from the same trial were included when they provided different or complementary biomarker analyses, clinical outcomes or follow-up data (e.g. when separate publications from the same trial reported distinct outcomes or additional biomarkers), while overlapping or superseded reports with early analyses or no additional data were excluded.

Reports were excluded if they (1) were not derived from a phase III randomized controlled trial; (2) evaluated phase I or II trials or non-randomized studies; (3) included localized disease, neoadjuvant and/or adjuvant treatment or systemic treatment beyond the first-line; (4) included exclusively non-clear-cell renal cell carcinoma (nccRCC); (5) did not evaluate an eligible ICI-based combination; (6) did not report a biomarker analysis; or (7) were protocols, reviews, editorials, commentaries, letters, case reports, preclinical studies or other publications not linked to randomized clinical trials. Duplicate, overlapping or superseded reports were excluded when they did not provide additional biomarker, outcome or follow-up data.

The review was restricted to biomarker analyses from first-line phase III randomized trials to ensure focus on contemporary first-line treatment selection and to reduce heterogeneity related to study design, treatment line, patient population and outcome assessment. Randomized phase III trials were also considered more suitable for exploring treatment-biomarker interactions, as the presence of a comparator arm facilitates, although does not definitively establish, the distinction between prognostic and predictive effects.

### Search strategy

An extensive literature search in PubMed, Web of Science and Scopus databases was performed using the above eligibility criteria, including reports published between 1 January 2018 and 18 October 2025. The choice of 2018 as the starting year for the search was related to the first publication of an immune combination in mRCC patients. The following keywords were used in our search strategy: “(first-line) and (renal cancer) and (phase III)”, “(first-line) and (renal cancer) and (phase III) and (biomarker)”. Furthermore, proceedings of the main international oncological congresses of American Society of Clinical Oncology (ASCO) Annual Meeting and Genitourinary Cancers Symposium, and European Society of Medical Oncology (ESMO), were searched from 2018 onwards for relevant abstracts. This search strategy was designed to maximize retrieval of secondary, exploratory and post hoc biomarker reports derived from first-line phase III trials, which are not consistently indexed under broader MeSH terms (such as “biomarkers” or “clinical trial” publications). Indeed, additional exploratory MeSH-based searches showed lower sensitivity and did not identify any further eligible reports.

Initial screening was performed by A.A.V. and M.M., and ineligible results were identified based on the titles and abstracts. If the study’s topic could not be ascertained from its title or abstract, the full-text version was retrieved for evaluation. Disagreement was resolved by discussion or consensus with other two co-authors (F.D.C. and M.C.C.).

### Data collection

Four investigators (F.D.C., M.C.C., M.M. and A.M.) independently extracted the following information from the included studies: first author’s name, year of publications, details of study population (number of total patients, number of patients with or without alterations analyzed, histotypes and proportion), study treatment, study design (endpoints), the molecular or circulating biomarkers investigated, laboratory methods used for the analyses, the correlations with outcomes in terms of hazard ratios (HR) and confidence interval (CI). Disagreement was resolved by discussion or consensus with A.A.V. Given the heterogeneity and non-comparability of the identified studies, evidence synthesis was performed using a descriptive narrative approach.

## Results

This systematic review included a total of 16 eligible reports assessing biomarkers in patients with mRCC treated with first-line ICI–based combinations in phase III randomized trials. Of these, 12 were full-text publications, while 4 were conference abstracts presenting post hoc exploratory analyses. Particularly, the reported data derived from the following phase III trials: IMmotion151 (IM151), CheckMate 214 (CM214), JAVELIN Renal 101 (JR101), KEYNOTE-426 (KN426), CLEAR, CheckMate 9ER (CM9ER), COSMIC-313 and ETER100 [10–17]. Most of the included studies focused on ccRCC and evaluated ICI–based combinations, including ICI–ICI and ICI–TKI regimens, compared with VEGF-targeted monotherapy, mostly sunitinib (SUN), with the exception of COSMIC-313, where the control arm was nivolumab plus ipilimumab (NIVO-IPI). Endpoints assessed across studies mainly included PFS and overall survival (OS), although interaction test for treatment effect was inconsistently reported.

Across studies, biomarker analyses were predominantly post hoc and exploratory; only a few were prespecified. Translational analyses were performed on different samples, including archival primary tumour tissue, metastatic tissue and peripheral blood, using a heterogenous range of platforms such as targeted DNA and RNA next-generation sequencing (NGS), whole-exome sequencing (WES), gene expression profiling (GEP), immunohistochemistry (IHC) and multiplex bead-based assay and electrochemiluminescence assay for peripheral blood biomarkers.

Detailed information on study populations, methods and sequencing technologies for each included study is reported in Supplementary Table 2.

Based on our research, we identified four main biomarker assessment approaches: IHC, RNA-sequencing, genomic sequencing and circulating biomarker analyses from peripheral blood. Among them, different groups of biomarkers were commonly tested: PD-L1 expression and CD8⁺ infiltration by IHC, gene expression signatures (e.g. immune, angiogenic, proliferation and metabolic signatures), genomic alterations (e.g. TMB) and key driver mutations and peripheral blood biomarkers (e.g., blood count, cytokines and kidney injury marker-1, KIM-1). A summary of main findings is reported in Supplementary Table 3, whereas key interpretation for each biomarker is summarized in Table 1.

**Table 1:** Summary of relevant biomarker associations evaluated in phase III randomized clinical trials of first-line ICI-based combinations in metastatic RCC stratified by platforms used

Biomarker	Trials	Key role
IHC		
PD-L1	*IM151 [17], CM214 [10], *JR101 [11], COSMIC-313 [15] and CM9ER [14], KN426 [12], CLEAR [13], ETER100 [16]	Broad ICI-based combos benefit regardless of PD-L1 PD-L1 + : negative prognostic value with SUN; potential predictive signal with NIVO-IPI
CD8⁺ tumour infiltration (density and topology)	JR101 [11], CM9ER [14]	High IM/inflamed CD8: potential predictive signal with ICI-based combos High tumour-centre CD8: positive prognostic value with SUN
RNA-sequencing/transcriptomic		
Immune-cell deconvolution	JR101 [11], CM214 [10]	High CD8 + T cells signature: potential predictive signal with AVE-AXI
Immune signatures (JAVELIN/IMmotion 150 Teff and myeloid)	JR101 [11], CM214 [10]	JAVELIN immune signature-high: potential predictive signal with AVE-AXI
TcellinfGEP/myeloid (mMDSC, gMDSC)	KN426 [12], CLEAR [13]	TcellinfGEP-high and mMDSC-high: potential predictive signal with PEM-AXI
Angiogenesis signatures	JR101 [11], CM214 [10], KN426 [12], CLEAR [13]	Angiogenesis-high: positive prognostic value and potential predictive signal with SUN
Proliferation/*MYC*	KN426 [12], CLEAR [13], CM214 [10]	Proliferation/MYC-high: negative prognostic value across all treatments
Metabolic signatures	JR101 [11], KN426 [12], CM214 [10], CM9ER [14]	Angiogenesis-related metabolic signatures: positive prognostic value with SUN; Oxidative phosphorylation/adipogenesis: potential predictive signal with NIVO-CABO Lipid metabolism: positive prognostic value across treatment arms
Clustering/molecular subtypes	IM151 [17], JR101 [11], KN426 [12], CLEAR [13], CM214 [10]/CM9ER [14]	Angiogenic clusters: favourable prognosis, SUN sensitivity Immune-proliferative clusters: greater benefit from ICI addition
Genomic sequencing		
TMB	JR101 [11] and CM214 [10]	No significant association
TIB	CM214 [10]	High TIB: positive prognostic value with SUN
HLA genotyping	JR101 [11], CM214 [10]	Allele-specific prognostic value with AVE-AXI and SUN
Driver mutations (*VHL*, *PBRM1*, *BAP1*, etc.)	IM151 [17], CM214 [10], JR101 [11], CLEAR [13], KN426 [12]	ICI-based combos benefit generally maintained across mutation groups *PBRM1* and VHL mutations: favourable prognosis/SUN sensitivity *CDKN2A/B, BAP1*, and *PTEN* mutation: mainly negative prognostic value
rDM (10-gene panel)	JR101 [11]	rDM-high: potential predictive signal for AVE–AXI vs SUN
Circulating biomarkers		
Blood counts	JR101 [11]	Low platelets/neutrophils, high lymphocytes; positive prognostic value across treatment arms
Peripheral T-cell metrics	JR101 [11]	High baseline T-cell fraction/number/clonality: potential predictive signal with SUN
Inflammatory cytokines	JR101 [11]	High IL-6, IL-10, CRP: negative prognostic value across arms
Serum KIM-1	CM214 [10], JR101 [11]	Lower baseline and early decline: positive prognostic value Dynamic changes: potential predictive signal with NIVO-IPI

*ATEZO-BEV* atezolizumab plus bevacizumab, *AVE-AXI* avelumab plus axitinib, *ccRCC* clear cell renal cell carcinoma, *CPS* combined positive score, *CRP* C-reactive protein, *HLA* human leukocyte antigen, *ICs* immune cells, *ICI* immune checkpoint inhibitor, *IHC* immunohistochemistry, *IL-6/8/10* interleukin 6/8/10, *IM* invasive margin, *IMDC* International Metastatic Renal Cell Carcinoma Database Consortium, *KIM-1* kidney injury molecule-1, *mMDSC/gMDSC* monocytic/granulocytic myeloid–derived suppressor cells, *mRCC* metastatic renal cell carcinoma, *NIVO-CABO* nivolumab plus cabozantinib, *NIVO-IPI* nivolumab plus ipilimumab, *ORR* objective response rate, *OS* overall survival, *PD-1* programmed cell death protein 1, *PD-L1* programmed death-ligand 1, *PEM-AXI* pembrolizumab plus axitinib, *PEM-LEN* pembrolizumab plus lenvatinib, *PFS* progression-free survival, *SUN* sunitinib, *TCs* tumour cells, *Teff* effector T-cells, *TIB* tumour inflammation burden, *TKI* tyrosine kinase inhibitor, *TMB* tumour mutational burden, *TME* tumour microenvironment

*Prespecified analyses; all other analyses should be considered exploratory

### Immunohistochemistry (IHC)

#### PD-L1 expression

The role of PD-L1 expression was investigated across several trials, although substantial heterogeneity existed in type of ICI, assays and scoring systems used, which evaluated staining on tumour cells (TCs) or tumour-infiltrating immune cells (ICs), and/or used the Combined Positive Score (CPS). Results from randomized studies suggested improved outcomes with ICI-based combinations in PD-L1 + disease, although this was not consistent across different studies.

In IM151, PD-L1 expression was evaluable in 178 patients in the atezolizumab-bevacizumab arm (ATEZO-BEV) and 184 in the SUN arm. Primary end point was PFS in patients with PD-L1 + disease. PD-L1 ICs ≥ 1% was associated with improved PFS with ATEZO-BEV compared with SUN (HR 0.74, 95% CI 0.57–0.96). PFS also showed a gradient of increasing benefit with increasing PD-L1 levels. However, independent radiology committee (IRC)–assessed PFS was no longer significant in the PD-L1 + population, and, surprisingly, patients with PD-L1 < 1% showed a trend towards better PFS compared with patients with PD-L1 + disease, likely due to divergence between IRC- and investigator-assessed PFS [17]. In the final analysis, no significant OS benefit was observed in either the intention-to-treat (ITT) or the PD-L1 + population [18]. In JR101 (*N* = 886 ITT), PD-L1 status was available for 802 patients, 397 receiving avelumab plus axitinib (AVE-AXI) and 407 receiving SUN. The two independent primary end points were PFS and OS among patients with PD-L1 + tumours. Unlike the IM151 study, no differential PFS benefit was observed according to PD-L1 status within AVE-AXI arm, although PD-L1 positivity was associated with shorter PFS in the SUN arm (HR 1.57, 95% CI 1.16–2.14, *p* < 0.01) [11].

In CM214, PD-L1 expression was assessed both on TCs and using the CPS. PD-L1 status was available for 992 patients (498 in the NIVO-IPI arm and 494 in the SUN arm). In the primary analysis, multivariable results suggested that baseline PD-L1 TCs ≥ 1% predicted poorer OS with SUN (PD-L1 < 1% vs ≥ 1%: HR 0.70, 95% CI 0.52–0.93, *p* = 0.01) but not with NIVO-IPI [19]. However, a subsequent exploratory biomarker analysis showed that in patients with PD-L1 TCs ≥ 1% and CPS ≥ 1, NIVO-IPI improved PFS compared with SUN (TCs: HR 0.43, 95% CI 0.31–0.60, *p* < 0.01; CPS: HR 0.68, 95% CI 0.56–0.82, *p* < 0.01), whereas no difference was observed for PD-L1 TCs < 1% TCs or CPS < 1 with similar PFS for NIVO-IPI and SUN. Within the NIVO-IPI arm, PD-L1 ≥ 1% TCs was associated with longer PFS compared with < 1% (HR 0.73, 95% CI 0.55–0.95, *p* = 0.02), while CPS was not. Associations remained significant after multiple testing correction. Similarly, in patients with PD-L1 ≥ 1% TCs and CPS ≥ 1, NIVO-IPI improved OS vs SUN (TCs: HR 0.57, 95% CI 0.40–0.82, *p* < 0.01; CPS: HR 0.72, 95% CI 0.57–0.89, *p* < 0.01). Among patients with PD-L1 TCs < 1% and CPS < 1, a smaller but significant OS benefit remained, suggesting that OS was improved with NIVO-IPI regardless of PD-L1 expression. Median OS within NIVO-IPI arm was similar between patients with PD-L1 TCs < 1% and ≥ 1% TCs, while it was shorter OS in CPS ≥ 1 compared with CPS < 1 (HR 1.40, 95% CI 1.06–1.87) [10].

In contrast to these findings, subgroup analyses from COSMIC-313 and CM9ER did not demonstrate any association between PD-L1 expression on TCs and survival or response outcomes [20]. Similarly, in exploratory analyses from KN426, CLEAR and ETER100, ICI-based combinations remained superior to SUN in terms of response and survival outcomes (PFS only for ETER100) regardless of PD-L1 expression assessed by CPS, suggesting no predictive or prognostic association, even when considered as a continuous variable [13, 16, 21]. An exception was observed in a subgroup analysis of KN426, where the PFS advantage of pembrolizumab plus axitinib (PEM-AXI) vs SUN was greater in patients with CPS ≥ 1 (HR 0.66, 95% CI 0.53–0.82). Furthermore, PD-L1 CPS as a continuous variable was negatively associated with OS in the SUN arm [12, 21].

#### CD8 + in tumour total area

In JR101, spatial organization of CD8 + tumour-infiltrating cells by IHC was assessed in the total tumour area, tumour centre and invasive margin (IM). Within the AVE-AXI arm, a larger IM area (dichotomised at the median) was associated with longer PFS (HR 0.59, 95% CI 0.36–0.97, *p* = 0.03). In contrast, in the SUN arm, greater CD8+ abundance within the tumour centre was associated with significantly longer PFS (HR 0.62, 95% CI 0.47–0.82, *p* < 0.01). Overall, no significant association between PFS and CD8+ density in any tumour region was observed within the AVE-AXI arm, although median PFS remained longer with the combination compared with SUN across corresponding CD8-defined subgroups, supporting that the clinical impact of baseline CD8+ infiltration may be modulated by treatment type [11].

CD8+ cell density and topology have also been evaluated in CM9ER. Higher CD8+ infiltration was associated with improved PFS with nivolumab plus cabozantinib (NIVO-CABO), but not with SUN. Among the 410 evaluable patients, CD8+ topology was associated with an inflamed phenotype and improved survival outcomes with NIVO-CABO versus SUN (*p *< 0.01 for PFS and OS). However, these associations were not confirmed in multivariable analyses [14].

### Transcriptomic signatures

Transcriptomic profiling across included trials was performed using heterogeneous platforms, although most studies employed bulk RNA sequencing (e.g. TruSeq RNA access or RNA-seq Illumina HiSeq), enabling derivation of gene expression signatures capturing angiogenic, immune-inflamed, stromal and proliferation profiles.

#### Immune cell deconvolution

In JR101, RNA signatures were available in 720 patients (350 in the AVE-AXI arm and 370 in the SUN arm). Deconvolution of 22 ICs within the tumour microenvironment showed that CD8 + T cells had a significant interaction test in relation to PFS in the combination arm, consistent with IHC findings and suggestive of potential predictive value. However, associations within each arm did not reach statistical significance [11]. In the CM214, no clear associations with survival outcomes were identified with immune-cell deconvolution [10].

#### Immune signatures

In IM151, the previously defined T-effector gene expression signature (Teff) from IM150 was retrospectively applied and showed that ATEZO-BEV improved PFS vs SUN in Teff–high tumours (HR 0.76, 95% CI 0.59–0.99) [22, 23].

In JR101, a 26-gene set immune signature (JAVELIN) was identified, including T-cell and natural killer (NK) signalling and inflammatory pathways. Patients with expression ≥ median had significantly longer PFS in the AVE-AXI arm (HR 0.60, 95% CI 0.44–0.83, *p* < 0.01), while no association was noted in the SUN arm. Evaluation of previously published immune signatures showed more limited associations: the IM150 Teff and myeloid-inflamed signature, either alone or combined, did not differentiate PFS in either arm [11]. Similar findings were observed in the CM214, where IM150-derived Teff and myeloid and JAVELIN signatures were not associated with PFS or OS in either treatment arm [10].

In the CLEAR and KN426, T-cell inflamed profile (TcellinfGEP) and myeloid populations (mMDSC, gMDSC) signatures were evaluated with heterogeneous findings. In the CLEAR study, these signatures did not meaningfully stratify the efficacy of pembrolizumab plus lenvatinib (PEM-LEN), and the PEM-LEN arm showed longer PFS than SUN arm regardless of signature subgroups. Within-arm analyses demonstrated that TcellinfGEP scores were positively associated with best objective response (BOR) in the SUN arm (*p* < 0.05), but not in the PEM-LEN arm. Nevertheless, ORR remained higher with PEM-LEN compared with SUN in the TcellinfGEP-high cohort (76.1% vs 47.8%), indicating maintained response regardless of immune signature [13]. In contrast, in KN426, higher TcellinfGEP and mMDSC signature scores were significantly associated with improved ORR, PFS and OS within the PEM-AXI arm, whereas no associations were observed in the SUN arm. Using a pre-specified cutoff, comparative analyses demonstrated improved PFS (HR 0.58, 95% CI 0.47–0.72) and OS (HR 0.77, 95% CI 0.61–0.96) with PEM-AXI vs SUN in the TcellinfGEP non-low subgroup. However, these associations were no longer significant after adjustments [21].

#### Angiogenesis

In IM151, high expression of the angiogenesis signature derived from IM150 was associated with improved PFS in the SUN arm (HR 0.59, 95% CI 0.47–0.75), whereas ATEZO-BEV improved PFS vs SUN in angiogenesis low tumours.

Overall, across JR101, KN426, CLEAR and CM214, angiogenesis signatures showed concordant findings, demonstrating a favourable association with outcomes in the SUN arm [11, 13, 21]. In JR101 and CM214, angiogenesis signatures were associated with improved PFS exclusively in the SUN arm and did not stratify outcomes in patients receiving AVE-AXI or NIVO-IPI [11]. However, in CM214, the association between angiogenesis score and PFS with SUN was not maintained after adjustment for multiple hypothesis testing [10]. Similarly, in KN426 and CLEAR study, angiogenesis signatures were positively associated with longer PFS in the SUN arm, despite the superiority of PEM-AXI and PEM-LEN was maintained across angiogenic strata [13, 21].

#### Proliferation and MYC

Overall, across KN426, CLEAR and CM214, proliferation and *MYC* signatures showed largely concordant results and were negatively and significantly associated with survival outcomes (PFS and/or OS), supporting a strong prognostic role [10, 13, 21]. In KN426, the proliferation signature in the PEM-AXI arm remained negatively associated with OS (*p* = 0.01) even after adjusting for TcellinfGEP, supporting a potential independent effect from the inflamed immune signature [21]. In CLEAR, *MYC* expression was negatively associated with response in the PEM-LEN arm, despite consistent superiority over SUN. Similarly, for time-to-event outcomes, superiority of PEM-LEN was observed across proliferation- and oncogenic pathway signatures (RAS and WNT) [13].

#### Metabolic signatures

In JR101, high expression of the oxygen transport signature was associated with longer PFS in both arms (AVE-AXI: HR 0.70, 95% CI 0.51–0.97, *p* = 0.03; SUN: HR 0.72, 95% CI 0.54–0.96, *p* = 0.02), consistent with a favourable prognostic role, although without evidence of treatment interaction [11]. Similarly, in KN426 the hypoxia signature was positively associated with ORR and OS within the SUN arm (*p* = 0.07 and *p* = 0.09, respectively), although this association was no longer significant after adjustment for the angiogenesis signature, likely due to biological overlap between hypoxia and angiogenic pathways [21].

In JR101, organic acid metabolism and glucocorticoid metabolism were associated with improved PFS in the SUN arm (HR 0.48, 95% CI 0.36–0.64, *p* < 0.01 and HR 0.73, 95% CI 0.54–0.99, *p* = 0.03, respectively), while no association was observed in AVE-AXI [11].

The lipid metabolism signature showed a favourable association with PFS in both arms, with a modest effect in AVE-AXI (HR 0.72, 95% CI 0.52–0.99, *p* = 0.05) and a stronger association in SUN arm (HR 0.53, 95% CI 0.41–0.69, *p* < 0.01). Similarly, in CM214, expression of transcripts related to adipogenesis and fatty acid metabolism was positively associated with OS in both the NIVO-IPI and SUN arms [10]. Comparable findings were found in CM9ER with the distinction that oxidative phosphorylation and adipogenesis signatures were associated with longer PFS and OS specifically in NIVO-CABO arm and not in SUN arm [14].

Finally, in JR101, Kyoto Encyclopedia of Genes and Genomes (KEGG) metabolic pathways (glycolysis, Krebs cycle, fatty acid metabolism, pentose phosphate and AMPK signalling) were also investigated but were not significantly associated with PFS in AVE-AXI despite trends for glycolysis and pentose phosphate. In contrast, Krebs cycle and AMPK pathways were significantly associated with PFS in the SUN arm [11]. In CLEAR, superiority of PEM-LEN was observed regardless of metabolic pathway activity, including glycolysis and hypoxia [13].

#### Human leukocyte antigen (HLA)

HLA may affect the presentation of antigens, potentially impacting response to ICIs [24]. In JR101, the potential contribution of antigen presentation pathways was explored through the *DUX4* gene expression signature, a transcriptional programme linked to reduced MHC class I expression. High *DUX4* expression was associated with a trend toward shorter PFS in the AVE-AXI arm, while no association was observed in the SUN arm [11].

#### Clustering

Different but broadly consistent transcriptomic molecular clusters were identified across trials, although predictive and prognostic implications remained heterogeneous. This clustering approach was first applied in IM151 by Motzer et al., providing a framework for subsequent analysis. In IM151, unsupervised transcriptomic analysis revealed seven molecular clusters with differential treatment outcomes: angiogenic/stromal (cluster 1), angiogenic (cluster 2), complement/Ω-oxidation (cluster 3), T-effector/proliferative (cluster 4), proliferative (cluster 5), stromal/proliferative (cluster 6) and snoRNA (cluster 7). Angiogenic/stromal and angiogenic clusters were associated with longer PFS regardless of treatment arm, whereas stromal/proliferative showed poorer outcomes, supporting potential prognostic relevance. Interestingly, the IMDC favourable risk group was predominantly represented in angiogenic tumours, while poor risk group showed a prevalence of immune/proliferative signature tumours (clusters 4, 5 and 6). Across treatment arms, no difference in PFS was observed between ATEZO-BEV and SUN in cluster 1, 2 and 3, with HR close to unity, indicating a limited benefit from ICI addition over angiogenesis blockade in these clusters. In contrast, a significant PFS benefit with ATEZO-BEV was observed in cluster 4 (HR 0.52, 95% CI 0.33–0.82) and cluster 5 (HR 0.47, 95% CI 0.27–0.82), supporting a benefit from ICI-based therapy in these clusters. A numerical trend toward improved PFS was also observed in cluster 6 with ATEZO-BEV. When biologically related clusters were grouped, an improved survival was observed for ATEZO-BEV in clusters 4 and 5 (HR 0.69, 95% CI 0.48–0.98) and in clusters 4, 5 and 7 combined (HR 0.70, 95% CI 0.50–0.98), supporting preferential benefit in immune-responsive phenotypes. Subsequent final OS analyses from the IM151 confirmed these patterns, with numerically longer OS in angiogenic clusters and shorter OS in proliferative clusters, regardless of the treatment. Improved OS was observed with SUN in the angiogenic cluster, and with ATEZO-BEV in cluster 4, 5 and 7, with a greater benefit when these clusters were combined (median OS, 35.4 vs 21.2; HR, 0.70; 95% CI, 0.50–0.98) [18].

In JR101, molecular clusters from IM151 were applied and showed similar molecular and clinical profiles. However, patients treated with AVE + AXI had improved PFS vs SUN regardless of molecular cluster, though the magnitude of improvement varied between clusters. Specifically, patients within the angiogenic/stromal (Cluster 1, HR: 0.55, 95% CI 0.31–0.97), complement/oxidation (Cluster 3, HR: 0.65, 95% CI 0.43–0.97) and T-effector/proliferative (Cluster 4, HR: 0.54, 95% CI 0.32–0.91) demonstrated significantly longer PFS with AVE + AXI compared to SUN [25]. Similarly to IM151, no significant difference in PFS was observed between AVE + AXI and SUN in cluster 2 (angiogenic).

In KN426 and CLEAR, molecular subtypes were defined using different transcriptomic approaches: IM151 signatures were applied in KN426, while CLEAR used previously published signatures derived from a study of pembrolizumab monotherapy across solid tumours [12, 26]. Nevertheless, molecular clusters were broadly comparable. Despite these differences, both trials consistently showed that ICI-based combinations were associated with superior ORR and PFS compared with SUN across all molecular subtypes [12, 13, 21]. Again, the magnitude of benefit was consistent with that observed in previous studies. Notably, in both trials, ORR was numerically lower in stromal/proliferative and proliferative subtypes across treatment arms, whereas within the SUN arm, ORR was higher in the angiogenic subtype [13, 21].

Similarly, in CM214 and CM9ER, despite the use of different clustering approaches, differences in PFS and OS between ICI-based combinations and SUN did not reach statistical significance when patients were stratified according to these clusters [10, 15]. However, in CM9ER, patients receiving NIVO-CABO had longer median PFS in the high IFN-γ signature tertile (*p* = 0.04) and shorter median PFS in the high EMT8 signature tertile (*p* = 0.01) [15].

### Genomic profiling

Genomic profiling was mainly performed using whole-exome sequencing (WES). In selected studies, additional approaches included TMB, targeted mutation panels (e.g. *VHL*, *PBRM1*, *SETD2*, *BAP1*) and HLA genotyping.

#### TMB and total indel burden (TIB)

In JR101 and CM214, TMB, defined as the total number of somatic missense mutations per tumour sample, was not associated with PFS or OS in either arm [10, 11].

In CM214, TIB, defined as the total number of small insertions and deletions detected by WES, was split at median (six indels) and showed no association with outcomes under ICI, whereas higher TIB was associated with improved outcomes in the SUN arm (PFS: HR 0.63, 95% CI 0.46–0.87, *p* < 0.01; OS: HR 0.70, 95% CI 0.49–1.01, *p* = 0.05). The association between TIB and PFS with SUN was also maintained after adjustment for multiple hypothesis testing [10].

#### HLA genotyping

In JR101, WES enabled identification of HLA alleles (≥ 5% prevalence) associated with outcomes. In the AVE-AXI arm, *HLA-A01:01* was associated with longer PFS (HR 0.64, 95% CI 0.42–0.97, *p* = 0.03), while *HLA-A03:01* was associated with shorter PFS (HR 1.44, 95% CI 1.01–2.06, *p* = 0.04). In the SUN arm, *HLA-B40:02* was associated with shorter PFS (HR 1.81, 95% CI 1.15–2.84, *p* = 0.03), whereas *HLA-B57:01* and HLA-C*06:02 were associated with longer PFS (HR 0.36, 95% CI 0.14–0.92, *p* = 0.03; HR 0.54, 95% CI 0.33–0.88, *p* = 0.01, respectively) [11].

In CM214, HLA genotyping was performed on HLA loci A, B and C, and patients were classified as heterozygous if all three loci were heterozygous and homozygous if one or more loci were homozygous. In this study HLA zygosity was not associated with outcomes in the NIVO-IPI arm [10].

#### Targeted gene alterations

Overall, somatic driver alterations in RCC showed heterogeneous but largely concordant patterns. The IM151 study showed that functional depletion of *PBRM1* or *KDM5C* was correlated with angiogenic transcriptional programmes, whereas loss of tumour suppressor genes including *CDKN2A/B* and *TP53* was aligned with proliferative clusters. In fact, according to clustering results, tumours harbouring *PBRM1* mutations showed favourable prognosis regardless of treatment. Patients treated with SUN harbouring *PBRM1* mutations showed longer PFS compared to those with non-mutant *PBRM1* (HR 0.67, 95% CI 0.51–0.87). Consistently, in KN426, *PBRM1* mutations were associated with longer OS within the SUN arm [18, 23]. In contrast, in *PBRM1* wt disease treatment was associated with longer PFS in ATEZO-BEV and NIVO-IPI arms vs SUN. However, in the CM214, this association was not retained after adjustment [10, 18, 23]. In contrast, *CDKN2A/B* alterations were associated with poorer prognosis but predicted benefit from ATEZO-BEV, with longer PFS (HR 0.63, 95% CI 0.41–0.96) vs SUN within the *CDKN2A/B*-mutant group. No difference in PFS was observed between treatments in the wild-type subgroup. Similarly, patients whose tumour harbours a loss-of-function alteration in *ARID1A* and *KMT2C* had a greater PFS with ATEZO-BEV (*ARID1A*: HR 0.50, 95% CI 0.26–0.96; *KMT2C*: HR 0.47, 95% CI 0.27–0.83) vs SUN [23], while outcomes were comparable between treatment arms in the corresponding wild-type groups.

In JR101, CLEAR and KN426, recurrent RCC driver mutations, including *VHL*, *PBRM1*, *SETD2*, *BAP1*, *MTOR*, *TP53* and *KDM5C*, showed limited or no consistent associations with survival outcomes or treatment interaction, and superiority of ICI-based combinations over SUN was maintained irrespective of mutational status [11, 13]. However, in KN426, in the SUN arm, *VHL* mutation was associated with longer OS, whereas *BAP1* mutation was associated with shorter OS [21].

A further panel of mutations revealed treatment-specific associations with PFS. In the AVE-AXI arm, *PTEN* mutations were associated with shorter PFS (HR 2.64, 95% CI 1.56–4.46, *p* < 0.01) but did not differentiate PFS in the SUN arm. Similarly, in CM214, exploratory analyses suggested that the subgroup with *PTEN* mutations showed unfavourable OS under NIVO-IPI [10, 11]. Moreover, in the AVE-AXI arm, several alterations correlated with longer PFS, including *CD163L1*, *DNMT1*, *IL16*, *MC1R*, *ABCA1*, *FOXO1*, *LOC728763/CROCC2*, *MYH7B*, *SPATA31C2* and *STAB2*. Conversely, *CD163L1* and *DNMT1* mutations were associated with worse outcomes in the SUN arm [11].

#### Others

In JR101, Fcγ receptor polymorphisms were investigated given the potential of avelumab to engage Fc receptors. These polymorphisms were not predictive of treatment benefit, although *FCGR3A* variants were associated with shorter PFS in the SUN arm (HR 1.53, 95% CI 1.15–2.02, *p* < 0.01) [11].

In the same study, WES identified a 10-gene double-mutant panel (rDM) including somatic mutations (*ABCA1*, *CD163L1*, *DNMT1*, *MC1R*, *MYH7B*, *STAB2*) and germline variants (*CROCC2/LOC728763*, *FOXO1*, *IL16*, *SPATA31C2*). rDM was associated with prolonged PFS in the AVE-AXI arm (HR 0.68, 95% CI 0.50–0.93, *p* = 0.02) but shorter PFS in the SUN arm (HR 2.42, 95% CI 1.60–3.67, *p* < 0.01), supporting a potentially predictive role. Interestingly, rDM and wild-type (wt) tumours also showed distinct tumour microenvironment and circulating immune profiles. Within rDM tumours, longer PFS was observed when memory B cells were high and naïve B cells were low. In contrast, within wt tumours, prolonged PFS correlated with high activated NK cells and low resting NK cells. Across both genomic subgroups, higher CD8 + T-cell infiltration was associated with longer PFS. Pretreatment tumour analyses also showed significant differences between rDM and wt tumours, including higher follicular helper T cells (*p* < 0.01) and M1 macrophages (*p* = 0.03). In rDM tumours, peripheral immune profiling showed lower leukocytes (*p* < 0.01), monocytes (*p* < 0.01) and neutrophils (*p* < 0.01), while circulating protein profiling showed lower eotaxin-1 (*p* = 0.01) and MCP-1 (*p* = 0.01), but higher MIP-1β (*p* < 0.01) and IL-7 (*p* < 0.01) [11, 27].

### Peripheral biomarkers

JR101 constitutes one of the largest integrative biomarker datasets in RCC. In the overall population, lower platelet (HR 1.48, 95% CI 1.24–1.76, *p* < 0.01), neutrophil (HR 1.38, 95% CI 1.16–1.65, *p* < 0.01), monocyte counts (HR 1.25, 95% CI 1.05–1.51, *p* = 0.02) and higher lymphocyte (HR 0.80, 95% CI 0.67–0.95, *p* = 0.01) and eosinophil counts (HR 0.81, 95% CI 0.68–0.98, *p* = 0.03) were associated with improved PFS. Within-arms analyses showed similar patterns: in the AVE-AXI and SUN arms, low platelets and low neutrophils were associated with longer PFS, whereas high lymphocytes were associated with better PFS in the SUN arm (HR 0.70, 95% CI 0.55–0.88, *p* < 0.01) [27].

Higher baseline peripheral T-cell metrics showed predictive value for benefit with SUN: higher fraction, number and clones of T cells in peripheral blood were associated with longer PFS (HR 0.73, 95% CI 0.57–0.94, *p* = 0.01; HR 0.67, 95% CI 0.52–0.86, *p* < 0.01; HR 0.76, 95% CI 0.57–0.99, *p* = 0.05, respectively). Furthermore, in the SUN arm, an increase vs baseline in peripheral T-cell quantitation measures was associated with longer PFS. Eventually, elevated inflammatory cytokines (IL-6, IL-10, and CRP) were negative prognostic factors for PFS, with significant interaction for IL-6 (*p* interaction = 0.04) and IL-10 (*p* interaction = 0.02), and a stronger association for IL-10 in the SUN arm [27].

Finally, in CM214, the role of serum KIM-1 was investigated and measured at baseline and at 3 weeks after first treatment. Across both arms, higher KIM-1 levels were associated with shorter OS independent of IMDC risk and tumour burden, and benefit for NIVO-IPI vs SUN was observed across KIM-1 tertiles. A decrease in KIM-1 from baseline was strongly associated with PFS and OS among patients treated with NIVO-IPI (median PFS and OS: 70.8 and 85.4 months vs 4.2 and 26.6 months for > 30% decrease vs > 30% increase) but not among patients treated with SUN [28]. Consistent findings were reported in JR101, in which lower baseline circulating KIM-1 and on-treatment decrease were associated with longer PFS and OS. Baseline KIM-1 levels were lower in angiogenic than in immune transcriptomic clusters [29].

## Discussion

In this systematic review, we collected evidence from molecular biomarker analyses derived from phase III randomized trials of first-line ICI-based combinations in mRCC, spanning IHC, transcriptomic signatures, genomic sequencing and circulating markers. Across trials and platforms, a consistent message emerges: while several biomarkers carry prognostic value, none reliably discriminates benefit between ICI-based regimens, or between combinations and TKI monotherapy. This partly reflects methodological heterogeneity, with largely post hoc, exploratory and underpowered analyses, and variable assays, cut-offs and tissue sources, that limit reproducibility and generalizability. Variability in the type of tissue analyzed (primary vs metastatic) may contribute to heterogeneity, given the potential differences in molecular profiles [30]. Yet beyond these technical constraints lies a biological one: the broad activity of ICI-based combinations may overcome baseline biological differences and dilutes the signal of any single predictive marker [13].

This tension is best exemplified by PD-L1. Despite being the most extensively investigated marker, PD-L1 behaved predominantly as a negative prognostic factor in the SUN arm and, only in CM214, as a modest predictive signal favouring NIVO-IPI. Across the remaining trials, the advantage of combinations was independent of PD-L1 status [10]. A systematic review and network meta-analysis by Quhal et al. showed that in patients with PD-L1 expression ≥ 1%, NIVO-IPI demonstrated a higher likelihood of improved PFS compared with other combinations [31]. However, PD-L1 may represent an incomplete proxy of complex immunologic processes, further confounded by cellular compartment assessed (tumour vs immune cells), antibody class (anti–PD-1 vs anti–PD-L1) and scoring method (TC vs IC vs CPS). In this context, combination regimens may reduce reliance on PD-L1 pathways, since TKIs may remodel tumour vasculature and myeloid compartments, while CTLA-4 blockade may expand T-cell priming [10, 32]. The ongoing CARE1 study is randomizing patients to NIVO-IPI or ICI-TKI according to PD-L1 expression to eventually test whether this marker can be used for treatment selection. Spatial information on immune microenvironment adds a further layer: CD8⁺ density at IM may reflect an “excluded” phenotype, in which abnormal vasculature prevents effective immune trafficking. The addition of a VEGFR-TKI can normalize tumour vasculature, improve immune cell access and enhance the activity of ICI-based combinations [11, 33].

Transcriptomic profiling may offer a more biologically anchored picture, suggesting that responses to VEGFR-TKIs and ICIs might differ according to specific cluster [23, 25, 34]. Angiogenic clusters and signatures were associated with favourable prognosis across treatment arms but limited additional benefit from ICIs, whereas immune/proliferative clusters and immune signatures derived the greatest advantage from ICI-based therapies [23, 25]. Similarly, the NIVOREN study showed that immune-inflamed clusters, characterized by high T-, B- and NK-cell infiltration and lower proportions of neutrophils, fibroblasts and endothelial cells, exhibited better outcomes with NIVO [35]. Again, in the CM-025 study, molecular subtypes characterized by overexpression of immune-related pathways, increased tumour-infiltrating immune cells, specifically enriched of CD8 +, PD-1 +, TIM-3-negative and LAG-3-negative cells, were associated with response and clinical benefit from NIVO [36, 37]. These findings suggest that the importance of transcriptomic clusters may extend beyond the first-line setting, reinforcing the consistency of these observations across distinct settings. However, in the era of ICI-TKI combinations, responders were observed across subgroups and prediction of treatment response appeared to be clearly not linear. Discordances across trials (anti–PD-1 versus anti–PD-L1 backbones, TKI versus bevacizumab, and differing clustering algorithms) and tumour heterogeneity (primary vs metastatic) represent a further limit. Nonetheless, BIONIKK and OPTIC RCC trials represent the first prospective demonstration that transcriptomic classification can potentially guide treatment selection in mRCC. In the biomarker-driven phase II BIONIKK trial, treatment allocation was guided by a 35-gene transcriptomic classifier that stratified patients into four biologically distinct subtypes (ccrcc1–4) [38]. Patients with angiogenic ccrcc2 tumours, enriched for favourable IMDC risk, showed the greatest benefit from VEGFR-TKIs, despite high response rates also to NIVO-IPI. Immune-desert ccrcc1 tumours had lower efficacy with both TKIs and nivolumab alone, although outcomes improved with NIVO-IPI. Conversely, immune-inflamed ccrcc4 subtypes, characterized by high PD-L1 expression, sarcomatoid features and poor prognosis, showed greater sensitivity to ICIs [38].

Similarly, in the OPTIC RCC phase II trial, patients with angiogenic clusters (1–2) received NIVO-CABO, whereas clusters 4–5 were treated with NIVO-IPI. Early data from the NIVO-CABO cohort showed high ORR, reduction of tumour burden in all patients and absence of primary refractory disease [39]. Given the limited benefit from the addition of ICIs in angiogenic clusters, it would be of interest to determine whether similar outcomes could be achieved with VEGF-TKI alone in this subgroup..

Unfortunately, genomic alterations showed limited predictive value. TMB was not predictive, consistent with RCC low mutation burden [40], and recurrent driver mutations (*PBRM1*, *VHL*, *CDKN2*, *PTEN*, *BAP1*) showed heterogeneous associations, behaving mainly as prognostic proxies of underlying angiogenic or proliferative biology rather than as predictors of differential treatment benefit. Interestingly, in JR101, *ABCA1* mutation, which encodes for a key cholesterol efflux transporter, leading to its reduced expression, was associated with prolonged PFS in the AVE + AXI arm [10]. Consistently, in the retrospective CHOMET study, higher tumour expression of *ABCA1* was associated with shorter survival. These findings suggest a potential role of cholesterol metabolism in modulating outcomes to ICI-based treatments, potentially paving the way to the exploration of the interplay between metabolism and immunity [41]. Eventually, a broader genomic profiling in JR101 identified a specific rDM phenotype associated with differential benefit from AVE-AXI. This phenotype exhibited a distinct immune microenvironment characterized by higher memory B cells, T helper cells, M1 macrophages, lower neutrophils and favourable cytokine profiles, thus correlating the immune architecture with the mutational burden. Genomic data, in other words, become meaningful when interpreted alongside the transcriptomic and immune context. Accordingly, future biomarker development should move from single-platform approaches towards integrated multi-omic models. Recently, proteogenomic profiling has revealed protein-level dysregulation of oxidative phosphorylation and metabolic pathways, not detectable at mRNA level alone, while also defining immune-based tumour subtypes with potential therapeutic relevance [42].

Peripheral biomarkers added a layer to this broad picture. Lower neutrophil and platelet counts and higher inflammatory cytokines (IL-6, IL-8, CRP) were strongly associated with worse outcomes, reflecting myeloid-driven immunosuppression and aggressive tumour biology, recapitulated by established prognostic models, such as IMDC and MEET-URO score [27, 43]. Similar results were drawn by the NIVOREN study, further supporting the role of circulating inflammatory biomarkers across different settings [35]. In this context, both baseline levels and on-treatment changes of KIM-1, a member of the TIM immune family, showed a consistent association with disease burden, early response to ICI-based therapies and survival outcomes. Post hoc analyses from CM214, COSMIC-313 and JR101 support an association between higher baseline KIM-1 levels and adverse clinical outcomes [28, 29, 44]. Recent evidence has further expanded its role across disease stages, including diagnosis, prognostication, minimal residual disease detection and treatment monitoring. In the adjuvant setting, analyses from IMmotion010 suggested that circulating KIM-1 may identify patients at increased risk of recurrence after nephrectomy and may help define a subgroup deriving greater benefit from adjuvant atezolizumab [45].

Similarly, circulating tumour DNA (ctDNA) should be acknowledged as an important but still immature circulating biomarker in RCC. In several solid tumours, ctDNA has shown clinical potential for molecular residual disease detection, relapse prediction and treatment monitoring; however, its translation to RCC has been slower partly because of low and heterogeneous ctDNA shedding and assay-dependent sensitivity [46]. A recent KEYNOTE-564 sub-analysis further underscored the limitations of current ctDNA implementation in RCC therapeutic scenario: after nephrectomy, baseline ctDNA positivity was very low, although specificity was high, and pembrolizumab benefit was observed regardless of baseline ctDNA status [47]. Biomarker development may be even more compelling in the adjuvant than in the metastatic setting, where the main challenge is to identify those patients who are most likely to benefit from adjuvant treatment while avoiding overtreatment of those already cured by surgery. Translating biomarker platforms developed in mRCC therefore represents a logical step toward more precise adjuvant treatment selection. The integration of molecular signatures with circulating biomarkers may outperform conventional pathological prognostic models, finally improving patient selection.

Multi-omic profiling has revealed substantial biological heterogeneity within disease-defined populations, dividing mRCC into increasingly small and biologically distinct subgroups. This challenges traditional “one-size-fits-all” trial models: indeed, it is unlikely that every biomarker–treatment combination can be evaluated in a separate conventional trial, thereby challenging traditional evidence-generation models. Biomarker-guided strategies may provide a more patient-centred framework. Among these strategies, umbrella trials, which test multiple biomarker-matched treatments within the same disease, and platform trials, which use an adaptive multi-arm, multi-stage structure in which interventions may be added or discontinued as evidence accumulates, could evaluate several biomarker–treatment hypotheses within a shared infrastructure, reducing resource requirements and enabling the incorporation of longitudinal tissue and circulating biomarkers as tumour biology evolves. Precision oncology therefore does not require abandoning evidence-based medicine, but rather broadening the ways in which evidence is generated through adaptive, patient-centred trial designs, instead of relying exclusively on a single rigid hierarchy of evidence [48, 49].

We acknowledge that our reviews have important limitations. Tissue-based biomarkers are challenged by tumour heterogeneity, temporal evolution and methodological variability. The delay between surgery and metastatic disease is variable and tumour characteristics might change, thus complicating interpretation. Many signatures and wide genomic testing are difficult to reproduce in routine clinical practice and to apply to a broader population, emphasizing the need for more accessible biomarkers and integrative approaches ^52^. Furthermore, small sample sizes of some subgroups and the lack of statistical power and/or consistent multiplicity adjustments for association analyses may hinder definitive conclusions. Trial differences in cut-offs, different algorithms for clustering patient samples and different gene expression signatures, limited comparative interpretations of biomarkers across trials. Finally, restricting eligibility to phase III randomized first-line trials may have excluded biologically relevant biomarker analyses from phase II studies in first- and later-line settings, which can provide important biological insights, and therefore considered for future validation in phase III trials. 

## Conclusion

Overall, associations between individual biomarkers and treatment outcomes were inconsistent and often exploratory across studies, reflecting biological complexity and methodological heterogeneity. Notably, ICI-based combinations often demonstrated superior efficacy over TKIs across most biomarker subgroups, suggesting that their therapeutic benefit may overcome baseline biological differences. At present, no biomarker can reliably guide treatment selection among available ICI-based combination regimens. Molecular stratification may not only inform the optimal choice of combination therapy, but also help identify patients who may benefit from therapeutic intensification or de-escalation. In this context, future efforts should increasingly focus on circulating biomarkers and integrated platforms, which can account for spatial and temporal tumour heterogeneity. Future biomarker research should increasingly move towards adaptive trial designs integrating longitudinal multi-omic profiling, which may provide more feasible strategies for generating clinical evidence.

## Supplementary Information

Below is the link to the electronic supplementary material.

ESM 1 (DOCX 29.2 KB)

ESM 2 (XLSX 21.7 KB)

ESM 3 (DOCX 19.2 KB)

## Data Availability

The data generated during and/or analysed during the current study are available from the corresponding author on reasonable request.
