# Supplementary material for: Molecular biomarkers in metastatic clear cell renal cell carcinoma treated with first-line immune combinations: a systematic review of phase III randomized clinical trials by Meet-URO group
Source: Cancer Metastasis Rev. 2026 Aug 26;45(3):64. doi: 10.1007/s10555-026-10371-w (PMC13518418; doi:10.1007/s10555-026-10371-w)
Supplement: Supplementary file 1 [file 10555_2026_10371_MOESM1_ESM.docx]

**Supplementary Table 1. Detailed search results, study-selection counts and reasons for exclusion**

| **Item** | **n** |
| --- | --- |
| PubMed/MEDLINE records | 226 |
| Web of Science records | 401 |
| Scopus records | 305 |
| Congress abstracts | 4 |
| Total database records | 936 |
| Duplicate records removed | 331 |
| Unique database records | 605 |
|  |  |
| Total records screened | 605 |
| Reports excluded by title and abstract | 565 |
| Reasons for exclusion: |  |
| - Wrong study design or trial phase | 143 |
| - Wrong treatment line or disease setting | 134 |
| - Wrong population or histology | 108 |
| - Review/guideline/meta-analyses/editorial/protocol/others papers | 98 |
| - No eligible biomarker analyses | 68 |
| - No first-line immune checkpoint inhibitors- based combinations evaluated | 14 |
| Reports after screening by title and abstracts | 40 |
|  |  |
| Reports assessed for eligibility | 40 |
| Reports excluded | 24 |
| Reasons for exclusion: |  |
| - Earlier/overlapping report superseded by a more updated follow-up and/or biomarker analysis | 19 |
| - No biomarker analyses | 5 |
| Reports included | 16 (12 full-text publications and 4 conference abstracts) |

PubMed was searched using two complementary strategies: “(first-line) and (renal cancer) and (phase III)”; “(first-line) and (renal cancer) and (phase III) and (biomarker)”. The first search retrieved 185 records, while the biomarker query retrieved 41 records, for a total of 226 records before deduplication. Duplicate records were identified using DOI and PMID matching, followed by verification of titles when these identifiers were not available
