# Supplementary material for: Molecular biomarkers in metastatic clear cell renal cell carcinoma treated with first-line immune combinations: a systematic review of phase III randomized clinical trials by Meet-URO group
Source: Cancer Metastasis Rev. 2026 Aug 26;45(3):64. doi: 10.1007/s10555-026-10371-w (PMC13518418; doi:10.1007/s10555-026-10371-w)
Supplement: Supplementary file 2 [file 10555_2026_10371_MOESM3_ESM.docx]

**Supplementary Table 3**. Summary of biomarkers evaluated in phase III randomized clinical trials of first-line ICI-based combinations in metastatic RCC stratified by platforms used

| Biomarker | Key findings | Treatment favored | Predictive vs prognostic | Prespecified vs exploratory |
| --- | --- | --- | --- | --- |
| IHC | | | | |
| PD-L1 | **IM151**, PD-L1 ICs ≥1% : ↑ PFS with ATEZO-BEV; no OS benefit.  **CM214**, PD-L1 TC ≥1% or CPS ≥1: ↑ PFS/OS with NIVO-IPI; OS benefit also present in PD-L1 low.  **JR101**: PD-L1 ICs ≥1% → ↓ PFS with SUN.  **COSMIC-313 & CM9ER**, PD-L1 TCs: no association.  **KN426, CLEAR, ETER100**: ICI-based combos superior vs SUN regardless PD-L1. **KN426**, PD-L1 CPS ≥1 → greater PFS benefit with PEM-AXI; PD-L1 CPS continuous: ↓ OS with SUN. | Broad ICI-based combos benefit regardless of PD-L1. NIVO-IPI in PD-L1+ | Negative prognostic (SUN); predictive (NIVO-IPI) | Prespecified: IMmotion151, JAVELIN101. Others exploratory/subgroup analyses |
| CD8⁺ tumour infiltration (density and topology) | **JR101,** larger IM CD8: ↑ PFS with AVE-AXI; higher tumour-centre CD8: ↑ PFS with SUN.  **CM9ER,** higher CD8 and inflamed CD8 topology: ↑ PFS/OS with NIVO-CABO (univariable only). | ICI-based combos; SUN (only tumour-centre CD8) | Predictive (ICI-based combos for IM CD8); prognostic (SUN, tumour-centre CD8) | Exploratory |
| RNA-sequencing / transcriptomic | | | | |
| Immune-cell deconvolution | **JR101,** CD8-PFS interaction with AVE-AXI; no within-arm association.  **CM214**: no association. | AVE-AXI | Predictive | Exploratory |
| Immune signatures (JAVELIN / IMmotion 150 Teff and myeloid) | **JR101,** JAVELIN signature ≥ median: ↑ PFS with AVE-AXI; Teff/Myeloid signatures: no association.  **CM214**: no association. | AVE-AXI | Predictive | Exploratory |
| TcellinfGEP / myeloid (mMDSC, gMDSC) | **KN426**, higher TcellinfGEP and mMDSC: ↑ ORR, PFS, OS with PEM-AXI.  **CLEAR**: no meaningful stratification; PEM-LEN superior regardless. | PEM-AXI | Predictive | Exploratory |
| Angiogenesis signatures | **JR101, CM214, KN426, CLEAR**: consistently ↑ PFS with SUN. | SUN | Predictive | Exploratory |
| Proliferation / *MYC* | **KN426, CLEAR, CM214,** high proliferation/*MYC*: ↓ PFS/OS. | None | Negative prognostic (ICI-based combos and SUN) | Exploratory |
| Metabolic signatures | **JR101, KN426,** oxygen transport, hypoxia, organic acid, glucocorticoid metabolism: ↑ PFS with SUN; lipid metabolism: ↑ PFS both arms.  **CM214,** adipogenesis/fatty acid metabolism: ↑ OS both arms.  **CM9ER,** oxidative phosphorylation/adipogenesis: ↑ PFS/OS with NIVO-CABO. | SUN (angiogenesis-related metabolism); NIVO-CABO (oxidative phosphorylation / adipogenesis); all arms (lipid metabolism) | Mostly prognostic | Exploratory |
| Clustering / molecular subtypes | **IM151,** angiogenic clusters: better prognosis; proliferative clusters: poor prognosis; clusters 1–3: no PFS difference between arms; clusters 4 and 5 and 4+5: PFS/OS benefit with ATEZO-BEV.  **JR101, KN426, CLEAR, CM214/CM9ER**: ICI-based combos superior across molecular subtypes, but variable magnitude of benefit. | ICI-based combos (immune clusters); SUN (angiogenic clusters) | Prognostic (angiogenic vs proliferative); predictive signal (ICIs addition in immune clusters) | Exploratory |
| Genomic sequencing | | | | |
| TMB | **JR101** and **CM214**: no association with PFS/OS. | None | None | Exploratory |
| TIB | **CM214,** high TIB: ↑ PFS with SUN. | SUN | Prognostic | Exploratory |
| HLA genotyping | **JR101,** in AVE-AXI arm: *HLA-A01:01*: ↑ PFS, *HLA-A03:01*: ↓ PFS; in SUN arm: *HLA-B40:02* ↓ PFS, *HLA-B57:01* and *HLA-C*06:02*: ↑ PFS.  **CM214**: HLA zygosity no association. | Allele-specific | Prognostic | Exploratory |
| Driver mutations (*VHL*, *PBRM1*, *BAP1*, etc.) | **IM151,** **CM214**: *PBRM1* mutations → better prognosis (better with SUN); *PBRM1* wt → favor ATEZO-BEV and NIVO-IPI arms. *CDKN2A/B* → poorer prognosis, benefit from ATEZO-BEV.  **JR101, CLEAR, KN426**: ICI-based combos superior regardless of mutation status. **KN426:** *VHL* → longer OS with SUN; *BAP1* → shorter OS with SUN.  **JR101,** *PTEN* mutation: shorter PFS with AVE-AXI.  **CM214,** *PTEN*: worse OS with NIVO-IPI. | Mutation-specific | Mainly prognostic | Exploratory |
| rDM (10-gene panel) | **JR101,** rDM: ↑ PFS with AVE-AXI; ↓ PFS with SUN. | AVE-AXI | Predictive | Exploratory |
| Circulating biomarkers | | | | |
| Blood counts | **JR101:** low platelets, neutrophils, high lymphocytes → better PFS. | AVE-AXI and SUN | Prognostic | Exploratory |
| Peripheral T-cell metrics | **JR101,** higher baseline T-cell fraction/number/clonality: ↑ PFS with SUN. | SUN | Predictive | Exploratory |
| Inflammatory cytokines | **JR101**: high IL-6, IL-10, CRP → worse PFS. | AVE-AXI and SUN | Prognostic | Exploratory |
| Serum KIM-1 | **CM214**: high baseline → worse OS; early decrease → improved PFS/OS with NIVO-IPI.  **JR101**: lower baseline and on-treatment levels → improved PFS/OS | NIVO-IPI, AVE-AXI | Prognostic and predictive | Exploratory |
